# Cognitive functioning in anxiety and depression: results from the ALSPAC cohort

**DOI:** 10.1098/rsos.221161

**Published:** 2023-08-09

**Authors:** Steph Suddell, Liam Mahedy, Caroline Skirrow, Ian S. Penton-Voak, Marcus R. Munafò, Robyn E. Wootton

**Affiliations:** ^1^ School of Psychological Science, University of Bristol, Bristol, UK; ^2^ National Institute for Health Research Biomedical Research Centre at the University Hospitals Bristol NHS Foundation Trust, University of Bristol, Bristol, UK; ^3^ MRC Integrative Epidemiological Unit, University of Bristol, Bristol, UK; ^4^ Population Health Sciences, Bristol Medical School, University of Bristol, Bristol, UK; ^5^ Cambridge Cognition, Cambridge, UK; ^6^ Nic Waals Institute, Lovisenberg Diaconal Hospital, Oslo, Norway

**Keywords:** anxiety, depression, cognition, response inhibition, emotion recognition, working memory

## Abstract

Anxiety and depression are associated with a range of impairments in cognitive functioning. Understanding the nature of these deficits may identify targets for intervention and prevent functional decline. We used observational and genetic methods to investigate the relationship of anxiety and depression with three cognitive domains: emotion recognition, response inhibition, and working memory, in the Avon Longitudinal Study of Parents and Children (ALSPAC). We examined: (i) cross-sectional associations between anxiety, depression, and cognition at age 24 (*n* = 2187), (ii) prospective associations between anxiety and depression at age 18 and cognition at age 24 (*n* = 1855), and (iii) the casual effect of anxiety and depression on cognition using Mendelian randomization (MR). Both disorders were associated with altered emotion recognition; anxiety with decreased happiness recognition (*b = −*0.27 [−0.54,0.01], *p* = 0.045), and depression with increased sadness recognition (*b =* 0.35 [0.07,0.64], *p* = 0.016). Anxiety was also associated with poorer working memory (*b = −*0.14 [−0.24,0.04], *p* = 0.005). There was no evidence for an association with response inhibition. MR provided no clear evidence of causal relationships between mental health and cognition, but these analyses were underpowered. Overall, there was little evidence for impairments in executive functioning, but moderate alterations in emotion recognition. This may inform the development of psychosocial interventions.

## 1. Introduction

Anxiety and depression are leading causes of disability worldwide [1]. The conditions are highly comorbid [2], with over 50% of depressed individuals also having an anxiety disorder [3]. Onset is common in adolescence and young adulthood, with earlier onset being associated with more severe trajectories [4,5]. In addition to the core symptoms of low mood and psychological distress, these conditions are often associated with poorer cognitive and psychosocial functioning [6] that persists even in remitted patients [7]. Understanding the nature of these deficits may identify targets for intervention and help prevent further functional decline.

To date, much of the research concerning mental health and cognitive functioning has been conducted in relatively small-scale psychological studies with heterogeneous designs, limiting comparisons across studies. However, meta-analyses synthesizing this work suggest that depressed individuals display moderate deficits in several domains, including attention, memory and processing speed [8–10]. While fewer meta-analyses have been conducted within anxiety disorders, those available also report deficits in episodic and working memory, learning and processing speed [11–14].

Anxiety and depression are also associated with differences in socio-cognitive domains, including altered emotional processing. However, previous research is inconsistent regarding the direction of effect. For example, both disorders have previously been associated with moderate to large deficits in facial emotion recognition [15–17], whereas a recent review of cognition in patients remitted from depression suggests their emotion recognition may actually be superior than that of healthy controls [9]. Further studies highlight that anxiety and depression are associated with emotional processing *biases*, with affected individuals displaying a tendency to interpret social information more negatively than healthy controls [15,18]. This may lead to an ‘uneven’ profile of recognition accuracy, varying by specific emotion.

However, as noted by de Nooij and colleagues [19], meta-analyses may overinflate effect sizes if the included studies rely on samples not reflective of a general population. In line with this, their investigation in the UK Biobank (aged 45–81 years), found that lifetime depression was associated with deficits in several domains of cognition, including executive functioning and processing speed, but effect sizes were smaller than in traditional case-control studies [19]. Earlier work, also in the UK Biobank (aged 40–69 years), found evidence for cognitive deficits associated with lifetime recurrent depression, but only in unadjusted analyses [20]. By contrast, research in the Generation Scotland study (average age 51 years [21]) reported lower processing speeds in depressed participants, in addition to superior vocabulary scores. These findings highlight the value of large, population-based, studies to estimate the relationship between cognitive functioning and mental health in mid-to-late adulthood. There is also a need to examine the relationship in young adulthood, when many cognitive functions reach maturity [22] and emotional disorders often emerge [4,5].

A further benefit of investigating mental health and cognition in population-based cohort studies is the opportunity to use genetically informed methods to strengthen causal inference. Mendelian randomization (MR) is one such method that estimates the causal relationship between two traits of interest, by using genetic variants to compare groups of individuals who differ on a phenotype of interest (such as mental health status or cognitive ability [23]). MR can reduce bias from some of the common pitfalls of observational methods such as reverse causation and residual confounding [24] and is increasingly being applied to study the aetiology of psychiatric conditions [25]. Applying MR in the present context can allow us to estimate the causal effect of anxiety and depression on a range of cognitive abilities.

The Avon Longitudinal Study of Parents and Children (ALSPAC) is a large birth cohort that presents an opportunity to study the relationship between cognition, anxiety and depression in adolescence and young adulthood using both observational and genetic epidemiology methods. In the present study, we examined the association between anxiety and depression with three domains of cognitive functioning in young adulthood: working memory, emotion recognition and response inhibition. We aimed to (i) examine the cross-sectional association between anxiety and depression and cognition at age 24, (ii) conduct prospective analyses to explore the relationship between anxiety and depression at age 18 and cognition at age 24, and (iii) triangulate this observational work with genetic analyses, using Mendelian randomization to strengthen causal inference.

## 2. Method

### 2.1. Participants

ALSPAC recruited pregnant women residing in the former county of Avon, UK, who were due to give birth between 1 April 1991 to 31 December 1992. An initial 14,541 pregnancies were enrolled, leading to 14,062 live births and 13,988 children being alive at 1 year. Data were collected at regular intervals via postal questionnaires and in-clinic assessments. The study population that completed the cognitive assessments at age 24 (*n* = 2187) used in this study is detailed in previous work [26] and in electronic supplementary material, tables S1 and S2. The study website contains details of all the data that are available through a fully searchable data dictionary and variable search tool (www.bristol.ac.uk/alspac). Any researcher can apply to use ALSPAC data, including the variables under investigation in this study.

Ethics approval for the study was obtained from the ALSPAC Ethics and Law Committee and the Local Research Ethics Committees. Written informed consent was obtained for the use of data collected via questionnaires and clinics from parents and participants following recommendations of the ALSPAC Ethics and Law Committee at the time. Consent for biological samples has been collected in accordance with the Human Tissue Act (2004). More details on ethics committees/institutional review boards are provided here: http://www.bristol.ac.uk/alspac/researchers/research-ethics/.

### 2.2. Measures

**Figure 1.**
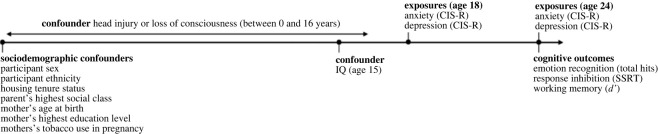
Timeline of variables used in the prospective and cross-sectional observational analyses. The outcome variables for all analyses were emotion recognition total hits (number of correctly identified facial expressions out of 96), response inhibition (as measured by SSRT: stop signal reaction time) and working memory (*d’*: *d* prime, a discriminability index on the *n*-back task) at age 24. Exposures were anxiety and depression at ages 18 and 24, as measured by the Clinical Interview Schedule (Revised; CIS-R).

A timeline of variables can be seen in figure 1. Study data were collected and managed using REDCap (Research Electronic Data Capture) which is a secure, web-based software platform supporting data capture in research hosted at the University of Bristol [27].

#### 2.2.1. Anxiety and depression

Anxiety and depression were assessed using the Revised Clinical Interview Schedule [28] (CIS-R) at both ages 18 and 24. The CIS-R is a self-administered computerized questionnaire which assesses a range of psychiatric symptoms to derive suggested diagnoses based on International Classification of Diseases, tenth revision (ICD-10) criteria for anxious and depressive disorders. It contains 14 sections, each assessing a different symptom over the past month and past week: sleep, fatigue, psychomotor agitation, anhedonia, self-harm, anxiety, memory and concentration, irritability, low mood, libido, feelings of guilt, specific phobia, depressive thoughts and panic symptoms. Any symptoms with scores of 2 or more are considered clinically significant. Participants rated each symptom on severity ranging from 0–4, with the exception of depressive symptoms which could be rated from 0–5. Where applicable, participants could also provide a reason for why they believed they had experienced a symptom (e.g. lack of sleep due to excessive noise) and the length of time they had been experiencing the symptom (less than two weeks, two weeks to six months, six months to 1 year, 1 to 2 years, or 2 years or more).

Two binary variables (present/absent) for suggested diagnoses of anxiety and depression were derived by aggregating scores across different symptom categories, in line with ICD-10 criteria. Participants had to have reported experiencing each symptom for over two weeks for it to be included in the score. The depression variable captured all individuals who met criteria for a primary or secondary suggested diagnosis of mild, moderate or severe depression. The anxiety variable captured all individuals who met criteria for a primary or secondary suggested diagnosis of generalized anxiety disorder, social phobia, agoraphobia, panic and non-specific neurotic disorders. This approach was taken to maximize sample size and to keep the operationalization of anxiety consistent with our genetic analyses, which used genetic variants associated with a combination of anxiety disorders [29].

#### 2.2.2. Cognitive outcomes

Participants completed three computer-based cognitive tasks (delivered using E-prime 2.0 Professional [30]) at age 24 years (*M* = 24.0, s.d. = 0.8). Full descriptions are in the electronic supplementary material. The distribution of the outcome variables from each cognitive task was assessed via boxplot, and outliers were excluded if they fell beyond 1.5 times the interquartile range. Normality was assessed at each level of the categorical anxiety and depression variables via histograms. Participants were excluded if they had not responded to a sufficient number of trials on each cognitive task. Exclusion of outliers, and due to poor responding, was only necessary for the working memory data (see description below).

**Emotion recognition.** An emotion recognition task (ERT) assessed accuracy in the recognition of facial displays of six emotions: happiness, sadness, anger, disgust, fear, and surprise. Participants were presented with a series of facial expression and asked to indicate which emotion had been displayed out of the six possible emotions. Stimuli ranged in intensity from a near-neutral expression to a prototypical display. The primary outcome measure of the ERT was total hits (the number of correctly identified facial expressions, out of 96). Secondary outcomes were the number of hits for each emotion category. Hits per emotion were out of 16, with higher scores indexing better emotion recognition.

**Working memory.** An *n*-back task was used as a continuous performance measure of working memory [31]. Participants monitored a series of numbers and indicated whether each number matched the one they saw two trials previously. Participants responded via keypress. The task consisted of 48 trials, eight of which were target trials (i.e. matches). The primary outcome measure was *d* prime (*d’*)*,* a discriminability index that takes into account the proportion of hits (correctly identified matches) to false alarms (non-matches incorrectly identified as matches) to estimate signal-detection ability [32]. Higher *d’* indicates better performance. Participants were excluded if they responded to fewer than 50% of trials or had a negative *d’*.

**Response inhibition.** A stop signal task [33] was used to assess participants' capacity to withhold a motor response. Participants were presented with a series of trials displaying either an ‘X’ or ‘O’ on a blank screen. On each trial, participants were asked to respond by pressing the corresponding key (X or O) as quickly as possible, unless they heard an auditory tone (the ‘stop signal’). Participants completed four blocks of 64 trials, 25% of which had a stop signal. The delay between stimulus onset and stop signal (stop signal delay) varied between trials. The primary outcome was stop signal reaction time (SSRT), calculated as the difference between the median reaction time for go trials and an estimate of the median stop signal delay (SSRT = Go Reaction Time_med_ – Median Stop Signal Delay). Median Stop Signal Delay was the latency where each participant was likely to fail to inhibit 50% of trials. Lower SSRTs indicate better response inhibition.

#### 2.2.3. Potential confounders

Adjustment was made for a range of sociodemographic variables previously found to be associated with mental health and cognitive outcomes. These were participant sex, highest parental social class (four levels using the 1991 Office of Population Census and Statistics Classification [34]: unskilled or semi-skilled manual, skilled manual or non-manual, managerial and technical, and professional), mother's highest education (determined during pregnancy and coded as below O-level, O-level, above O-level, indicating completion of a school-leaving qualification at age 16), housing tenure (owned/mortgaged versus other), maternal tobacco use during pregnancy (present/absent), mother's age at birth, and child ethnicity (non-white/white). Confounders relating to cognition were IQ and head injury. IQ was assessed with the Wechsler Abbreviated Scale of Intelligence (vocabulary and matrix reasoning tests; [35]) at age 15, which was the nearest available timepoint to the exposure. Head injury was defined as a cracked skull or loss of consciousness at any timepoint from 0 to 16 years (coded as present/absent), collected via parent- or self-report.

### 2.3. Statistical analyses

Primary analyses were conducted in R [36]. R scripts for the observational and genetic analyses are openly available in the electronic supplementary material.

#### 2.3.1. Observational analyses

We used multivariable linear regression to examine the association between anxiety and depression and the three primary cognitive outcome measures (ERT total hits, SSRT and *d’*). A second multivariable model examined the association between anxiety and depression and individual emotions on the ERT (hit scores for happiness, sadness, anger, disgust, fear and surprise). All models were conducted cross-sectionally (exposure: anxiety and depression assessed at 24 years) and prospectively (exposure: anxiety and depression assessed at 18 years).

Anxiety and depression were initially entered as exposures in separate models. Each model was then adjusted for (i) sociodemographic variables (sex, ethnicity, parental occupation, mother's education, housing tenure, mother's age at birth and mother's tobacco use in pregnancy); (ii) additionally, history of head injury and IQ; and (iii) concurrent anxiety or depression at that timepoint, to determine if there was a unique effect of either mental health exposure. The final model at each timepoint was the same for both anxiety and depression.

We initially conducted complete case analyses, using participants with data for all exposures, outcomes and confounders (cross-sectional *n* = 2187; prospective *n* = 1855). As missing data can lead to biased estimates [37], we conducted multiple imputation using the ‘ice’ package in Stata [38]. We included a range of auxiliary variables, in addition to all exposure and confounders, to predict missing data and impute 100 datasets. Exposure and confounder data were imputed for all participants who had completed all three cognitive assessments (*n* = 3087). We present the multiply imputed analyses as the primary results. A comparison of available and missing ALSPAC participants is presented in electronic supplementary material, table S1.

Finally, we conducted exploratory analyses to examine the effect of symptom duration on cognition. Here we stratified anxiety and depression cases into two groups based on whether they reported experiencing depressive/worry symptoms for shorter or longer than six months. This was entered as an exposure in a linear regression model, predicting cognitive functioning at age 24.

#### 2.3.2. Genetic analyses

We conducted Mendelian randomization (MR) to examine possible causal pathways between cognition and anxiety and depression. We initially sought to conduct MR bidirectionally, using genetic instruments for both cognition and anxiety/depression. However, genome-wide association studies (GWAS) of the three primary cognitive outcomes in ALSPAC did not yield any genome-wide significant single nucleotide polymorphisms (SNPs) to use as genetic instruments [26]. Therefore, we conducted MR analyses in the direction of mental health exposure to cognitive outcome, using published summary statistics from anxiety and depression GWAS as exposures. Analyses were conducted using the TwoSampleMR package 0.4.26 [39].

For depression MR analyses, we used 40 SNPs associated with major depression previously identified by Wray and colleagues [40] comparing 135 458 cases and 344 901 controls. For anxiety, we used summary statistics from a meta-analysis of GWAS of anxiety disorders performed by Otowa and colleagues (case-control analysis, total *n =* 17 310; [29]). One genome-wide significant SNP was identified. We therefore used a relaxed threshold of *p* < 5 × 10^−5^, which identified 497 SNPs. These SNPs were clumped at linkage disequilibrium (LD) *r*^2^ = 0.001 and a distance of 10 000 kb using the *clump_data* command prior to analysis, resulting in 87 independent loci.

We sought to conduct both one- and two-sample MR. For one-sample, we generated polygenic risk scores (PRS) of anxiety and depression for each participant using PLINK (v. 1.90; [41]), which summed the number of risk alleles for each SNP weighted by the effect estimate of that SNP in the discovery GWAS. We then ran logistic regressions, regressing each PRS onto anxiety/depression at age 24 in ALSPAC, to confirm that the PRS were internally valid. We then aimed to conduct instrumental variable regressions, regressing the residuals from the PRS to anxiety/depression analyses onto cognitive outcome measures in our ALSPAC sample. For two-sample MR, we compared results across three methods: inverse-variance weighted (IVW), weighted median [42] and weighted mode [43]. IVW is the primary method, and each of the others are sensitivity tests that make different assumptions regarding the validity of the genetic instruments [42,43]. A consistent effect across all methods would provide the most robust evidence for a causal effect.

#### 2.3.3. Interpretation of evidence

When interpreting the results of the statistical analyses, we have avoided using terms such as ‘statistically significant’ to describe effects. This approach was taken following recent discussions on the topic [44]. Instead, we transparently report effect estimates, confidence intervals and exact *p*-values. We consider the strongest evidence for an effect to be a precise confidence interval around an estimate, which remains in a consistent direction with each level of adjustment, alongside evidence for a causal effect in the MR analysis.

## 3. Results

### 3.1. Anxiety and depression

A breakdown of the sample by anxiety and depression case/control status, alongside summary cognitive scores, can be seen in electronic supplementary material, table S2. At age 18, 7.2% of the complete cases met ICD-10 criteria for a primary or secondary diagnosis of depression, 8.5% met the criteria for a primary or secondary diagnosis of an anxiety disorder and 3.8% of the sample met criteria for both. At age 24, 9.5% of complete cases met criteria for depression and 12.0% met criteria for an anxiety disorder, while 6.2% met criteria for both anxiety and depression. Of the participants with data at both timepoints (*n* = 1847), around a third of anxiety and depression cases at age 18 were also symptomatic at age 24 (33% and 29%, respectively; electronic supplementary material, table S3). The majority of participants who did not meet criteria for anxiety and depression at age 18 also did not meet criteria at age 24 (90% and 92%, respectively).

### 3.2. Cross-sectional analyses

At age 24, there was evidence for a negative association between anxiety and working memory (*d’*, table 1). This was consistent across all levels of adjustment. There was no clear evidence for an association between depression and working memory. No clear evidence was found for an association between either anxiety or depression and response inhibition (SSRT).

**Table 1.** Cross-sectional and prospective associations with emotion recognition, working memory and response inhibition (imputed dataset). ERT: emotion recognition task total hits; SSRT: stop signal reaction time; *d*’: *d* prime, a discriminability index on the *n*-back task.

			unadjusted			model 1			model 2			model 3		
time	outcome	exposure	*b* [95% CI]	*p*	$R_{\mathrm{adj}}^{2}$	*b* [95% CI]	*p*	$R_{\mathrm{adj}}^{2}$	*b* [95% CI]	*p*	$R_{\mathrm{adj}}^{2}$	*b* [95% CI]	*P*	$R_{\mathrm{adj}}^{2}$
cross-sectional	emotion recognition (ERT)	depression	0.19 [−0.69,1.08]	0.673	0.000	0.21 [−0.67,1.08]	0.642	0.042	0.25 [−0.61,1.11]	0.575	0.104	0.23 [−0.78,1.25]	0.654	0.104
		anxiety	0.48 [−0.32,1.28]	0.240	0.00	0.38 [−0.41,1.18]	0.342	0.042	0.14 [−0.64,0.91]	0.342	0.104	0.02 [−0.89,0.94]	0.962	—
	response inhibition (SSRT)	depression	5.55 [−0.56,11.65]	0.075	0.001	2.76 [−3.35,8.87]	0.376	0.021	2.62 [−3.46,8.71]	0.398	0.032	0.24 [−6.96,7.45]	0.947	0.032
		anxiety	5.86 [0.34,11.39]	0.037	0.001	3.43 [−2.09,8.95]	0.223	0.021	4.14 [−1.37,9.65]	0.141	0.033	4.02 [−2.50,10.55]	0.227	—
	working memory (*d’*)	depression	−0.06 [−0.15,0.03]	0.201	0.000	−0.03 [−0.12,0.07]	0.592	0.034	−0.02 [−0.11,0.07]	0.611	0.077	0.06 [−0.05,0.16]	0.284	0.079
		anxiety	−0.11 [−0.20,−0.03]	0.008	0.002	−0.09 [−0.17,0.00]	0.044	0.035	−0.11 [−0.19,−0.03]	0.008	0.079	−0.14 [−0.24,−0.04]	0.005	—
prospective	emotion recognition (ERT)	depression	1.03 [−0.08,2.14]	0.069	0.000	0.91 [−0.19,2.01]	0.106	0.043	0.97 [−0.10,2.04]	0.075	0.105	0.73 [−0.48,1.95]	0.237	0.105
		anxiety	0.88 [−0.13,1.89]	0.088	0.000	0.83 [−0.17,1.83]	0.105	0.043	0.82 [−0.17,1.80]	0.105	0.105	0.52 [−0.61,1.65]	0.367	—
	response inhibition (SSRT)	depression	8.33 [0.49,16.18]	0.037	0.000	5.42 [−2.45,13.30]	0.177	0.022	5.24 [−2.59,13.07]	0.189	0.033	3.86 [−4.86,12.57]	0.385	0.033
		anxiety	7.26 [0.01,14.51]	0.050	0.000	4.55 [−2.70,11.80]	0.218	0.022	4.56 [−2.64,11.76]	0.214	0.033	3.00 [−5.00,11.00]	0.463	—
	working memory (*d’*)	depression	−0.12 [−0.24,−0.01]	0.038	0.000	−0.09 [−0.21,0.02]	0.115	0.035	−0.09 [−0.20,0.03]	0.130	0.078	−0.08 [−0.21,0.05]	0.229	0.078
		anxiety	−0.08 [−0.19,0.03]	0.168	0.000	−0.05 [−0.16,0.06]	0.349	0.034	−0.06 [−0.16,0.05]	0.317	0.077	−0.02 [−0.14,0.10]	0.701	—

*Note. n* = 3087 in 100 multiply imputed datasets. Model 1: adjusted for participant sex, ethnicity, housing tenure, parent's highest social class, mother's age at birth, mother's tobacco use in pregnancy, mother's highest education level; Model 2: additionally adjusted for IQ at age 15 and head injury by age 16; Model 3: additionally adjusted for concurrent anxiety or depression at time of exposure.

There was also no clear evidence for an association between anxiety or depression with global emotion recognition (ERT total hits, table 1); however, effect estimates were consistently positive. When analysing individual emotions (table 2), there was some evidence that both anxiety and depression were associated with poorer recognition of happy faces. However, the evidence for the association with anxiety was consistently stronger, and in the final model (including both anxiety and depression), only the anxiety effect remained. By contrast, there was strong evidence for a specific association between depression and an increased recognition of sad faces, which was consistent across all models, where anxiety effects did not survive adjustment for depression. There was no clear evidence for associations with the recognition of any other emotions. Complete case analyses are presented in electronic supplementary material, tables S4–S6.

**Table 2.** Cross-sectional associations with emotion-specific hit rate on the emotion recognition task (imputed dataset).

		unadjusted			model 1			model 2			model 3		
emotion	exposure	*b* [95% CI]	*p*	$R_{\mathrm{adj}}^{2}$	*b* [95% CI]	*p*	$R_{\mathrm{adj}}^{2}$	*b* [95% CI]	*p*	$R_{\mathrm{adj}}^{2}$	*b* [95% CI]	*p*	$R_{\mathrm{adj}}^{2}$
happy	depression	−0.19 [−0.43, 0.06]	0.135	0.000	−0.27 [−0.51, −0.02]	0.034	0.017	−0.27 [−0.51, −0.02]	0.035	0.017	−0.11 [−0.40, 0.19]	0.478	0.018
	anxiety	**−**0.22 [−0.45, 0.00]	0.049	0.001	−0.32 [−0.54, −0.09]	0.005	0.018	−0.32 [−0.55, −0.10]	0.005	0.018	−0.27 [−0.54, −0.01]	0.045	—
sad	depression	0.35 [0.11, 0.60]	0.005	0.002	0.38 [0.14, 0.63]	0.002	0.026	0.39 [0.15, 0.63]	0.002	0.067	0.35 [0.07, 0.64]	0.016	0.066
	anxiety	0.28 [0.06, 0.50]	0.014	0.002	0.29 [0.07, 0.51]	0.010	0.026	0.23 [0.01, 0.45]	0.039	0.065	0.06 [−0.20, 0.32]	0.655	—
anger	depression	**−**0.07 [−0.36, 0.21]	0.625	0.000	−0.06 [−0.34, 0.23]	0.690	0.020	−0.05 [−0.33, 0.24]	0.751	0.061	−0.08 [−0.41, 0.26]	0.656	0.061
	anxiety	0.07 [−0.19, 0.33]	0.588	0.000	0.08 [−0.18, 0.33]	0.568	0.020	0.01 [−0.24, 0.27]	0.914	0.061	0.05 [−0.25, 0.35]	0.741	—
disgust	depression	0.03 [−0.23, 0.29]	0.816	0.000	0.03 [−0.23, 0.29]	0.843	0.010	0.03 [−0.23, 0.29]	0.820	0.024	−0.09 [−0.39, 0.22]	0.581	0.025
	anxiety	0.21 [−0.02, 0.45]	0.075	0.001	0.19 [−0.04, 0.43]	0.109	0.011	0.16 [−0.08, 0.39]	0.194	0.025	0.20 [−0.08, 0.48]	0.164	—
surprise	depression	**−**0.14 [−0.31, 0.04]	0.117	0.000	−0.13 [−0.31, 0.04]	0.144	0.009	−0.13 [−0.30, 0.05]	0.155	0.011	−0.12 [−0.33, 0.08]	0.243	0.011
	anxiety	**−**0.06 [−0.22, 0.09]	0.425	0.000	−0.06 [−0.22, 0.10]	0.453	0.009	−0.07 [−0.23, 0.09]	0.416	0.011	−0.01 [−0.19, 0.18]	0.951	—
fear	depression	0.21 [−0.19, 0.60]	0.305	0.000	0.26 [−0.14, 0.65]	0.202	0.024	0.27 [−0.12, 0.66]	0.179	0.055	0.27 [−0.19, 0.73]	0.248	0.055
	anxiety	0.21 [−0.15, 0.56]	0.256	0.000	0.20 [−0.15, 0.56]	0.260	0.024	0.12 [−0.23, 0.48]	0.492	0.055	−0.01 [−0.42, 0.41]	0.970	—

*Note. n* = 3087 in 100 multiply imputed datasets. Model 1: adjusted for participant sex, ethnicity, housing tenure, parent's highest social class, mother's age at birth, mother's tobacco use in pregnancy, mother's highest education level; Model 2: additionally adjusted for IQ at age 15 and head injury by age 16; Model 3: additionally adjusted for concurrent anxiety or depression at time of exposure (age 24).

Within the suspected depression cases, we found evidence for a relationship between the duration of symptoms and multiple cognitive outcomes (electronic supplementary material, table S7). Participants who reported experiencing depression symptoms for longer than six months demonstrated poorer emotion recognition and working memory ability than those experiencing symptoms for fewer than six months. Within anxiety cases, there was no clear relationship between self-reported duration of anxiety symptoms and any of the cognitive outcomes. The observed effects in the depression cases remained after adjusting for all sociodemographic and cognitive covariates (Model 2).

### 3.3. Prospective analyses

There was some evidence that individuals with depression at age 18 performed worse on both the response inhibition and working memory tasks, and better on emotion recognition ability (table 1). However, these associations attenuated after adjustment. There was no clear evidence for a prospective association between anxiety at age 18 and any of the three primary cognitive outcomes.

When analysing individual emotion hits (table 3), there was evidence that both depression and anxiety at age 18 were positively associated with recognition of fearful faces at age 24. These associations were consistent until adjusting for concurrent anxiety and depression. Anxiety at age 18 was also found to have a consistent, positive association with the recognition of disgusted faces at age 24. There was no clear evidence for associations with the recognition of any other emotions.

**Table 3.** Prospective associations with emotion-specific hit rate on the emotion recognition task (imputed dataset).

		unadjusted			model 1			model 2			model 3		
emotion	exposure	*b* [95% CI]	*p*	$R_{\mathrm{adj}}^{2}$	*b* [95% CI]	*p*	$R_{\mathrm{adj}}^{2}$	*b* [95% CI]	*p*	$R_{\mathrm{adj}}^{2}$	*b* [95% CI]	*p*	$R_{\mathrm{adj}}^{2}$
happy	depression	**−**0.08 [−0.39, 0.22]	0.597	0.000	−0.17 [−0.48, 0.13]	0.271	0.017	−0.17 [−0.48, 0.14]	0.274	0.016	−0.10 [−0.44, 0.24]	0.556	0.017
	anxiety	**−**0.13 [−0.41, 0.16]	0.375	0.000	−0.19 [−0.48, 0.09]	0.186	0.017	−0.19 [−0.48, 0.09]	0.188	0.017	−0.15 [−0.47, 0.17]	0.351	—
sad	depression	0.14 [−0.17, 0.44]	0.384	0.000	0.14 [−0.17, 0.44]	0.385	0.024	0.15 [−0.15, 0.45]	0.328	0.064	0.19 [−0.14, 0.53]	0.258	0.064
	anxiety	**−**0.01 [−0.30, 0.28]	0.938	0.000	−0.01 [−0.30, 0.28]	0.942	0.024	−0.02 [−0.30, 0.27]	0.909	0.064	−0.09 [−0.41, 0.22]	0.556	—
anger	depression	0.10 [−0.26, 0.45]	0.593	0.000	0.08 [−0.27, 0.43]	0.657	0.020	0.10 [−0.25, 0.44]	0.583	0.061	0.04 [−0.35, 0.43]	0.847	0.061
	anxiety	0.14 [−0.18, 0.47]	0.384	0.000	0.14 [−0.18, 0.47]	0.385	0.020	0.14 [−0.17, 0.46]	0.378	0.062	0.13 [−0.23, 0.49]	0.486	—
disgust	depression	0.35 [0.03, 0.67]	0.033	0.000	0.33 [0.00, 0.65]	0.047	0.012	0.34 [0.02, 0.66]	0.040	0.026	0.17 [−0.19, 0.53]	0.355	0.028
	anxiety	0.44 [0.14, 0.73]	0.004	0.003	0.43 [0.13, 0.73]	0.004	0.014	0.43 [0.13, 0.72]	0.005	0.028	0.36 [0.02, 0.69]	0.036	—
surprise	depression	0.08 [−0.13, 0.30]	0.434	0.000	0.09 [−0.13, 0.30]	0.432	0.009	0.09 [−0.12, 0.30]	0.417	0.011	0.10 [−0.13, 0.34]	0.394	0.011
	anxiety	0.00 [−0.21, 0.21]	0.993	0.000	0.01 [−0.20, 0.22]	0.948	0.009	0.01 [−0.20, 0.22]	0.932	0.011	−0.03 [−0.27, 0.20]	0.782	—
fear	depression	0.45 [−0.03, 0.92]	0.065	0.000	0.45 [−0.02, 0.93]	0.062	0.025	0.47 [0.00, 0.94]	0.049	0.056	0.33 [−0.21, 0.86]	0.227	0.056
	anxiety	0.44 [−0.01, 0.88]	0.053	0.001	0.45 [0.00, 0.89]	0.048	0.025	0.44 [0.00, 0.88]	0.048	0.056	0.31 [−0.19, 0.81]	0.227	—

*Note. n* = 3087 in 100 multiply imputed datasets. Model 1: adjusted for participant sex, ethnicity, housing tenure, parent's highest social class, mother's age at birth, mother's tobacco use in pregnancy, mother's highest education level; Model 2: additionally adjusted for IQ at age 15 and head injury by age 16; Model 3: additionally adjusted for concurrent anxiety or depression at time of exposure (age 18).

### 3.4. Mendelian randomization

For one-sample MR analyses, neither the anxiety or depression PRS predicted the corresponding phenotype in ALSPAC (anxiety: OR = 1.08, 95% CI = 0.96 to 1.20, *p* = 0.823; depression: OR = 1.03, 95% CI = 0.91 to 1.17, *p* = 0.607). Therefore, we were unable to continue this analysis past the validation stage.

Two-sample MR results are presented in table 4. There was very weak evidence for a possible effect of depression on improved global emotion recognition (IVW estimate: 0.275, 95% CI: −0.04 to 0.56, *p* = 0.082), with a consistent direction of effect across sensitivity analyses. There was no evidence for causal effects in any of the remaining MR models.

**Table 4.** Two sample Mendelian randomization analyses of mental health and cognitive outcomes. ERT: emotion recognition task total hits; SSRT: stop signal reaction time; *d*’: *d* prime, a discriminability index on the *n*-back task.

		depression (*n* SNPs = 40)		anxiety (*n* SNPs = 72)	
outcome	method	Beta [95%CI]	*p*	Beta [95%CI]	*p*
emotion recognition (ERT total hits)	inverse-variance weighted	0.275 [−0.035, 0.585]	0.082	−0.006 [−0.054, 0.042]	0.803
	weighted median	0.141 [−0.282, 0.563]	0.513	−0.016 [−0.088, 0.056]	0.658
	weighted mode	0.007 [−0.884, 0.897]	0.988	−0.036 [−0.170, 0.098]	0.601
response inhibition (SSRT)	inverse-variance weighted	0.014 [−0.332, 0.361]	0.935	−0.010 [−0.058, 0.038]	0.682
	weighted median	0.168 [−0.276, 0.613]	0.458	0.024 [−0.048, 0.096]	0.513
	weighted mode	0.429 [−0.465, 1.323]	0.353	0.053 [−0.086, 0.193]	0.457
working memory (*d’)*	inverse-variance weighted	0.082 [−0.213, 0.377]	0.586	−0.027 [−0.075, 0.022]	0.282
	weighted median	−0.029 [−0.456, 0.398]	0.893	0.005 [−0.069, 0.079]	0.888
	weighted mode	−0.650 [−1.602, 0.302]	0.189	0.081 [−0.084, 0.246]	0.341

## 4. Discussion

We investigated the relationship of anxiety and depression with three domains of cognition (emotion recognition, working memory and response inhibition) in a population sample of young adults. Cross-sectionally, we observed a negative association between anxiety and working memory performance, as well as both anxiety and depression being associated with differences in emotion recognition accuracy. There was also evidence for prospective associations between anxiety and depression at age 18 and increased recognition of negative emotions at age 24. We sought to triangulate these findings with genetic analyses; however, MR results were inconclusive, due to limited statistical power. This study is one of few cohort studies examining cognition and mental health in young adulthood, a critical time point for the development of mental health disorders [4,5]. Our results suggest that, while there is some evidence for deficits in executive functioning, these differences are small. However, individuals with anxiety and depression display moderate differences in emotion recognition, which has the potential to affect their daily psychosocial functioning. Over time, even small cognitive deficits may lead to enduring functional impairments such as poorer social relationships, unemployment, and, in later life, are thought to be vulnerability factors for dementia [45].

Emotion recognition ability has received attention as both a potential biomarker and causal mechanism in emotional disorders [46]. Contrary to some previous research [16], we did not find evidence for a general deficit in global emotion recognition ability. Although the evidence was weak, effect estimates were consistently positive. This trend remained consistent even when controlling for potential confounding variables such as participant IQ. There was, however, some evidence that symptom duration might moderate this relationship as, among suspected depression cases, participants who reported experiencing symptoms for longer than six months had poorer emotion recognition than those experiencing symptoms for shorter periods.

When studying recognition accuracy by emotion, we found strong evidence that both depression and anxiety were associated with a more ‘negative’ pattern of responding. Cross-sectionally, depression was associated with an increased accuracy of recognizing sad faces, while anxiety was associated with a decreased accuracy of recognizing happy faces. A similar pattern was identified prospectively, with both anxiety and depression being associated with superior recognition of fearful faces, and anxiety with recognition of disgust. It is unclear why the response pattern (in terms of the specific negative emotion being recognized) varied across timepoints. However, these findings are consistent both with previous research suggesting that depression is associated with a negative bias [47], and previous research suggesting that individuals who have experienced a depressive episode may actually have superior emotion recognition relative to controls [48]. Taken together, our results provide support that emotional disorders in young adulthood are associated with aberrant emotional processing. While further research is required to determine the generalizability of these findings to real-life social interactions, a small but consistent bias towards recognizing negative emotions could aggregate to large effects in an individuals' social experience.

In terms of executive functioning, there was evidence that, cross-sectionally, anxiety was associated with mild impairments in working memory performance. Previous research has suggested deficits in both anxiety [11,49] and depression [50]. However, we found weaker evidence for an association with depression. A potential explanation for this disparity is that the present research investigated depression in a general population sample, whereas previous research has often relied on sampling procedures that may result in larger effect sizes (such as recruiting depressed individuals from primary care services). In partial support of this, our exploratory analyses of the relationship between working memory and symptom duration suggested that depression cases who had been experiencing symptoms for longer had poorer working memory ability. Furthermore, previous research has often studied anxiety or depression in isolation, despite their high diagnostic comorbidity [2]. This makes it unclear whether previously identified effects in depression were primarily driven by anxiety symptoms.

There was also no clear evidence that anxiety and depression at age 18 were prospectively associated with either working memory or response inhibition at age 24. While some evidence was found in unadjusted analyses (both cross-sectionally and prospectively), this attenuated when adjusting for key sociodemographic and cognitive variables. Such variables are often unavailable in smaller psychological studies of cognition, highlighting the importance of longitudinal research in this area. Overall, our findings are in line with some previous work suggesting that the effect of anxiety and depression on executive functioning is likely to be modest [19,51].

This study has several limitations. First, it is likely that the relationship between cognition and mental health is bidirectional, and many cognitive models of emotional disorder posit that altered cognitive functioning precedes the development of psychological symptoms [46,52]. Unfortunately, we were unable to conduct analyses in the direction of cognition to mental health, due to (i) no subsequent mental health phenotypic data being available and (ii) no valid genetic instruments for the cognitive measures studied here. Nonetheless, this work is an important first step in investigating cognition in ALSPAC, which can be built upon as further clinic data becomes available. To address a lack of genetic instruments for cognition, future work could meta-analyse cognition across cohort studies to improve power. Second, our MR analyses (in the direction of mental health to cognition) also suffered from low power, leading to imprecise estimates, and the depression and anxiety PRS did not predict the corresponding phenotypes in our sample—preventing one-sample MR analyses. Third, while evidence suggests that anxiety and depression symptoms lie on a continuum [53], we relied on discrete variables and, for the anxiety variable, we included participants who met criteria for a range of anxiety disorders. This meant it was not possible to assess the effect of symptom severity, nor the potential burden of comorbid anxiety disorders. The CIS-R also does not record history of anxiety or depression, meaning it is unclear how age at onset or how multiple recurrences of illness might impact cognitive functioning. Finally, it was beyond the scope of the current study to evaluate the mechanism underlying the relationship between mental health and cognition. Future research should investigate the role of psychiatric comorbidity, such as psychosis and substance use disorder, which have also been found to contribute to deficits in cognitive functioning [54,55].

There was little evidence that anxiety and depression in a general population sample were associated with significant impairments in executive functioning. However, there were moderate associations with emotion recognition accuracy, with both disorders being associated with increased accuracy in recognizing more negative emotions. This may inform the development of interventions that target psychosocial functioning in emotional disorders.

## Data Availability

R scripts for the observational and genetic analyses reported in this article are available in the electronic supplementary material [56]. The informed consent obtained from ALSPAC participants does not allow the data to be made freely available through any third party maintained public repository. However, data used for this submission can be requested from the ALSPAC Executive. Full instructions for applying for data access can be found here: http://www.bristol.ac.uk/alspac/researchers/access/. The ALSPAC study website contains details of all the data that are available (http://www.bristol.ac.uk/alspac/researchers/our-data/).
